# A New Method for the Determination of Nucleic Acid Using an Eu^3+^– nicotinic Acid Complex as a Resonance Light Scattering Probe

**DOI:** 10.3390/molecules14010010

**Published:** 2008-12-23

**Authors:** Meng Guo, Lin-Tong Wang, Xia Wu, Wei Xu, Jing-He Yang

**Affiliations:** 1 Microscale Science Institute, Department of Chemistry, Weifang University, Weifang 261061, P.R. China. E-mail:guomeng7101@126.com (M. G.); 2 Key Laboratory of Colloid and Interface Chemistry (Shandong University), Ministry of Education, School of Chemistry and Chemical Engineering, Shandong University, Jinan 250100, P.R. China

**Keywords:** Nucleic acid, Nicotinic acid, Eu^3+^, Resonance light scattering.

## Abstract

This study found that in Tris-HCl buffer, the resonance light scattering (RLS) intensity of the Eu^3+^-nicotinic acid system can be greatly enhanced by nucleic acids and the enhanced intensity is proportional to the concentration of nucleic acid in the range of 7×10^-8^-1×10^-5 ^g∙mL^-1^ for fsDNA, and its detection limit is 2×10^-8 ^g∙mL^-1^. Based on this, a new method for the determination of nucleic acids is proposed. Synthetic and actual samples are determined satisfactorily. The interaction mechanism is also studied. It is thought that nucleic acid can bind with the Eu^3+^-nicotinic acid complex through electrostatic attraction and thus form a large Eu^3+^-nicotinic acid-nucleic acid complex.

## Introduction

Nucleic acids have an important function in life processes, so they are often used as a reference for measurements of other components in biological samples. However, direct use of the intrinsic fluorescence and ultraviolet absorption of nucleic acids for their determination has been severely limited by low sensitivity [1]. As a result some probes based on the interactions between nucleic acids and extrinsic reagents have been employed in the determination of nucleic acids using spectral methods [2]. Among these spectral methods, the resonance light scattering (RLS) technique has seen strong interest among chemists and biochemists since Pasternack *et al*.’s pioneering work [3] using a common spectrofluorometer. Later on, Huang *et al*. established a new method to determine the nucleic acid content by the RLS technique [4,5]. This method has been used extensively in recent years. Up to now, most of the probes applied in the determination of nucleic acids are organic dyes [2,6,7,8,9,10,11,12,13], thanks to their aggregation on DNA, whereas the use of metal ion complexes as RLS probes is rare [14,15]. To the best of our knowledge, there are no reports on the study of nucleic acids using the Eu^3+^-nicotinic acid complex as an RLS probe. In this study, it was found that nucleic acids could enhance the RLS intensity of Eu^3+^-nicotinic acid. Based on this, a new method for the determination of nucleic acid is proposed. The interaction mechanism is also discussed.

## Results and Discussion

### RLS spectra of the systems

**Figure 1:**
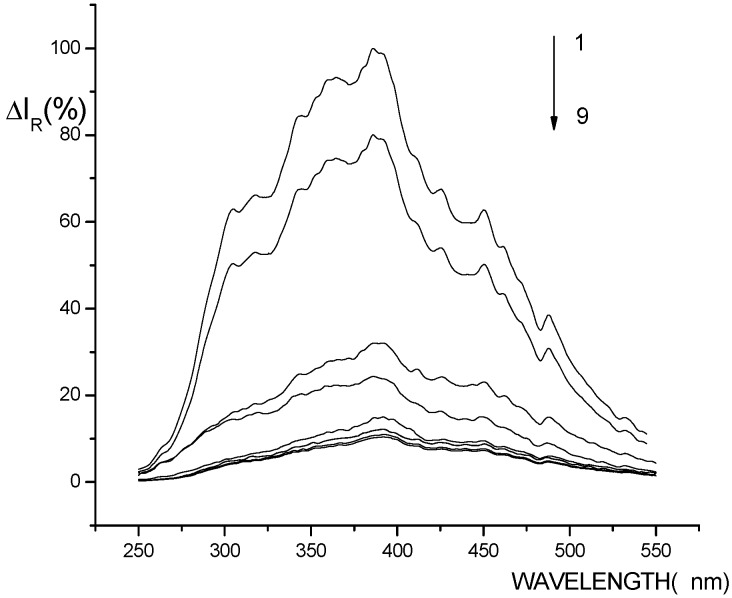
Apparent RLS profiles of Eu^3+^-nicotinic acid with DNA and RNA.

The apparent RLS spectra of Eu^3+^–nicotinic acid-fsDNA (1), Eu^3+^–nicotinic acid-yRNA (2), Eu^3+^–nicotinic acid (3), Eu^3+ ^(4), nicotinic acid (5), fsDNA (6), yRNA (7), nicotinic acid- fsDNA (8) and nicotinic acid- yRNA (9) systems are shown in Figure 1. It can be seen that in Tris–HCl buffer (pH 8.1), all the systems have the same RLS peak at about 387 nm. But the RLS intensity of the Eu^3+^–nicotinic acid system can be enhanced by nucleic acids, which indicates that there is the interaction between nucleic acid and Eu^3+^–nicotinic acid complex.

(1) Eu^3+^-nicotinic acid-fsDNA; (2) Eu^3+^-nicotinic acid-yRNA; (3) Eu^3+^-nicotinic acid; (4) Eu^3+^; (5) nicotinic acid; (6) fsDNA; (7) yRNA; (8) nicotinic acid-fsDNA; (9) nicotinic acid- yRNA. Conditions used were nicotinic acid: 5.0×10^-7 ^M; Eu^3+^: 2.8×10^-4^ M; Tris-HCl (0.05 M): pH=8.1; nucleic acids (fsDNA, yRNA): 1.0×10^-6^ g∙mL^-1^.

### Optimization of conditions for RLS measurements

**Figure 2:**
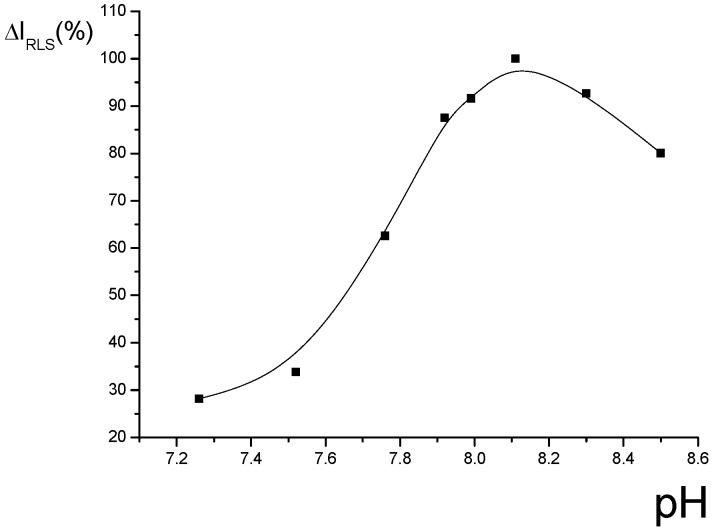
Effect of pH on RLS intensity of Eu^3+^-nicotinic acid-fsDNA.

*The effects of pH and buffer solution:* The effect of pH on the ΔI_R_ of the system was tested and is shown in Figure 2. The results indicate that the ΔI_R_ reaches a maximum when the pH is about 8.1, so this value was fixed for further research. The effect of the buffers on the ΔI_R_ of this system are shown in Table 1. The results indicate that different buffers also have large effect on the ΔI_R_ of the system, and 0.55 ml of Tris-HCl ( 0.05 M, pH=8.1) is the most suitable buffer.

Conditions: nicotinic acid: 5.0×10^-7 ^M; Eu^3+ ^: 2.8×10^-4^ M; fsDNA: 1.0×10^-6^ g∙mL^-1^.

**Table 1:** The choice of buffer solutions.

Buffers	Tris-HCl	HMTA^*^-HCl	NaH_2_PO_4_-K_2_HPO_4_	NH_4_Cl-NH_3_	NaH_2_PO_4_-Citric acid
ΔI_R_（%）	100	50.9	12.8	45.9	49.3

* HMTA- Hexamethylene tetramine; Conditions used were pH: 8.10; nicotinic acid: 5.0×10^-7 ^M; fsDNA: 1.0×10^-6 ^g∙mL^-1^; Eu^3+^: 2.8×10^-4 ^M.

**Figure 3:**
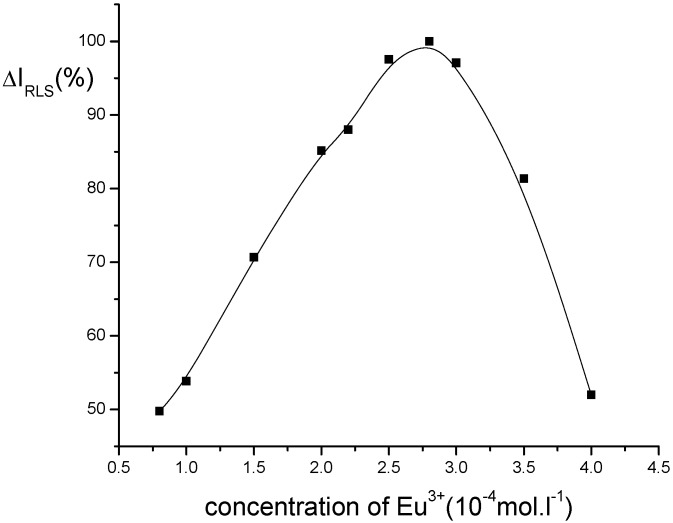
Effect of Eu^3+^ concentration on RLS intensity of Eu^3+^-nicotinic acid-fsDNA.

*The choice of rare earth ions and the effect of Eu^3+ ^concentration:* Different rare earth ions can influence the RLS intensity of the system, and the results are shown in Table 2. It is indicated that Eu^3+ ^is suitable for this system. The effect of Eu^3+ ^concentration on the ΔI_R_ of this system is shown in Figure 3. It can be seen that the ΔI_R_ is the strongest when the concentration of Eu^3+ ^is 2.8×10^-4 ^M. Therefore, 2.8×10^−4^ M Eu3+ was chosen for this research.

**Table 2:** Effect of different rare earth ions on RLS intensity of Eu^3+^-nicotinic acid-fsDNA.

M^3+^	Eu^3+^	Tb^3+^	Y^3+^	Nd^3+^	Al^3+^	Dy^3+^	Gd^3+^
I_R_(%)	100	46.4	99.0	54.6	66.6	68.2	65.0

Conditions: Tris-HCl (0.05 M): pH=8.1; nicotinic acid: 5.0×10^-7 ^M; fsDNA: 1.0×10^-6 ^g.mL^-1^; M^3+^: 2.8×10^-4 ^M.

Conditions: nicotinic acid: 5.0×10^-7 ^M; Tris-HCl(0.05 M): pH=8.1; fsDNA: 1.0×10^-6^ g∙mL^-1^.

**Figure 4:**
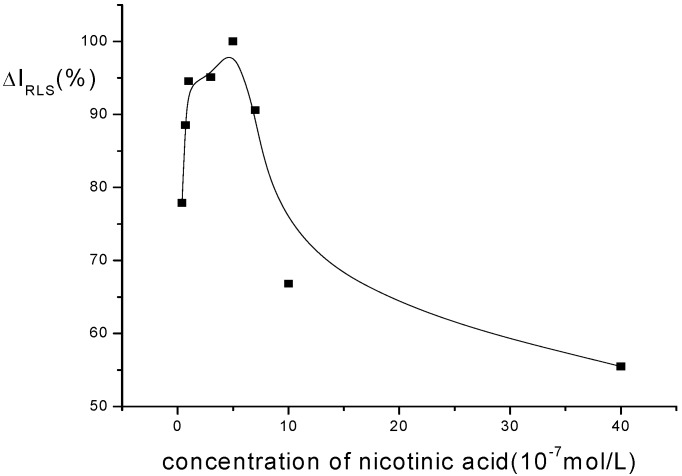
Effect of nicotinic acid concentration on RLS intensity of Eu^3+^-nicotinic acid-fsDNA.

*The effect of nicotinic acid concentration:* The effect of nicotinic acid concentration on the ΔI_R_ is shown in Figure 4. It can be seen that the ΔI_R_ of the system reaches the maximum when the concentration of nicotinic acid is 5.0×10^-7 ^M. Thus, 5.0×10^-7 ^M nicotinic acid is chosen in this research.

Conditions: Eu^3+^: 2.8×10^-4^ M; Tris-HCl(0.05 M): pH=8.1; fsDNA: 1.0×10^-6^ g∙mL^-1^.

*Addition order and stability of this system:* The effect of the addition order on the ΔI_R_ was tested. The result indicates that the order of nicotinic acid, fsDNA, Eu^3+ ^and Tris-HCl buffer is the best. Under the optimum condition, the effect of time on the fluorescence intensity is studied. The result shows that the ΔI_R_ of the system reaches a maximum within 15 min after all the reagents are added and remains stable for 3 h.

*Effect of foreign substances:* The interference of foreign substances is tested for 1.0×10^-6^ g∙mL^-1 ^fsDNA. The results show that within ±5% relative error, 92-fold of NaCl, 60-fold of KCl and CuCl_2,_ 35-fold of MgCl_2 _do not interfere with to the determination of fsDNA. Fourteen-fold ZnSO_4_, 7-fold CaCl_2_, Al(NO_3_)_3_, dextrose do not interfere with the determination of fsDNA. Two-fold FeCl_3_, citric acid is an obvious interferent in the determination of fsDNA.

### Analytical application

*Calibration curve and detection limit:* Under the optimum conditions defined here, the calibration graph for nucleic acid was obtained. The result indicates that there is a linear relationship between the ΔI_R_ of this system and the concentration of fsDNA in the range from 7.0×10^-8^-1.0×10^-5^ g∙mL^-1^, and the detection limit (S/N=3) is 2.0×10^-8 ^g∙mL^-1^. A comparison with other RLS probes is shown in Table 3. Although the sensitivity of this probe for the determination of nucleic acid is not high, it has lower toxicity. 

**Table 3:** Different probes of nucleic acid by RLS.

Probe	Nucleic acid	Detection limit (10^-9^g∙mL^-1^)	References
TAPP^*1^	ctDNA/fsDNA/yRNA	4.1/4.6/6.7	[4]
Safranine T	ctDNA/fsDNA/yRNA	13.2/39.8/61.1	[5]
Neutral Red	ctDNA/fsDNA/yRNA	48.2/35.2/205	[6]
BCB^*2^	ctDNA/fsDNA/yRNA	118/112/434	[7]
Methyl Green	ctDNA/fsDNA/yRNA	7.8/2.6/9.9	[9]
Azur A	ctDNA/fsDNA	19.9/12.6	[11]
Crystal Violet	ctDNA/fsDNA/yRNA	13.8/36.8/69	[8]
Methyl Violet	DNA	100	[10]
Congo Red	fsDNA/ctDNA/yRNA	0.019/ 0.89/1.2	[12]
Methyl green	fsDNA	2.6/7.8/9.9	[13]
OA^*3^–Eu^3+^	fsDNA/ctDNA/yRNA	0.02/0.011/0.01	[14]
Eu^3+^-TTA-Phen	fsDNA/yRNA/ctDNA	0.03/0.006/0.002	[15]

1. TAPP: a,b,g,d-tetrakis [4-(trimethylammoniumyl) phenyl] porphine; 2. BCB: brilliant cresol blue; 3. OA: oxolinic acid.

*Sample determination:* Considering the effects of foreign substances on the RLS intensity of the system, the standard addition method was used for the determination of DNA in synthetic and cucumber samples. The synthetic sample contained fsDNA (1.0×10^-6 ^g/mL), Na^+ ^(4.0×10^-6 ^g/mL), K^+ ^(2.0×10^-6 ^g/mL), Mg^+ ^(2.0×10^-6 ^g/mL) and Cl^- ^(1.0×10^-5 ^g/mL). The result indicates that the recovery ratio and relative standard deviation of this method are 97.3% and 4.7%, respectively. At the same time, cucumber samples were also determined. The recovery ratio was 107.8% and the relative standard deviation is 4.7%. So the accuracy and precision of this method are satisfactory.

**Figure 5:**
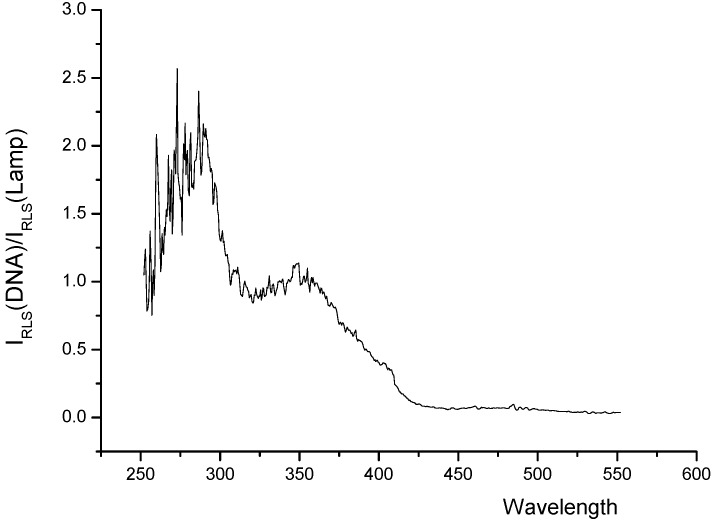
The corrected RLS profiles of Eu^3+^-nicotinic acid-fsDNA system.

**Figure 6:**
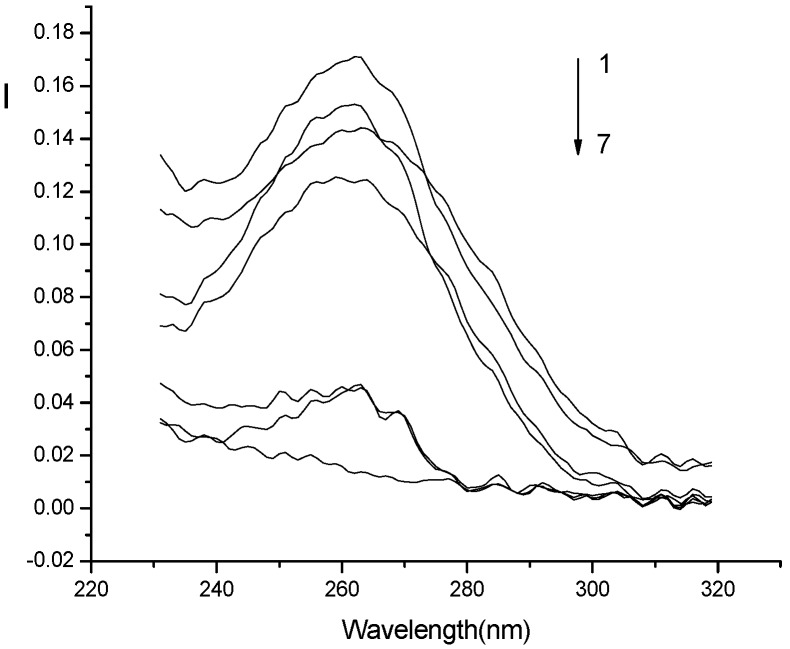
Absorption spectra.

*Interaction mechanism:* The light-scattering spectrum not only depends on the nature of the system, but also reflects the characteristics of the instrument [16]. In order to eliminate the effect of the instrument on the light scattering spectrum, the corrected light-scattering spectra of the systems are recorded by using the correction technique [17] and is shown in Figure 5. It can be seen from this figure that the scattering peak of the Eu^3+^-nicotinic acid-fsDNA system at about 260 nm is located within the absorption bands of both nicotinic acid and fsDNA in the range of 240–280 nm (Figure 6). Therefore, the light scattering of the studied system is ascribed to the resonance light scattering [3], and is considered to be due to the synergetic resonance caused by the absorption of both nicotinic acid and fsDNA.

Lamp and Eu^3+^-nicotinic acid-fsRNA. Conditions used were nicotinic acid: 5.0×10^-7 ^M; Eu^3+ ^: 2.8×10^-4^ M; Tris-HCl (0.05 M): pH=8.1; nucleic acids (fsDNA, yRNA): 1.0×10^-6^ g∙mL^-1^.

(1) nicotinic acid-Eu^3+^-fsDNA; (2) nicotinic acid-fsDNA; (3) Eu^3+^-fsDNA; (4) fsDNA; (5) nicotinic acid-Eu^3+^; (6) nicotinic acid; (7) Eu^3+^. Conditions used were nicotinic acid: 5.0×10^-7 ^M; Eu^3+^: 2.8×10^-4 ^M; Tris-HCl (0.05 M): pH=8.1; nucleic acids (fsDNA): 1.0×10^-6 ^g∙mL^-1^.

Figure 1 also shows that when Eu^3+^ is added to nicotinic acid system, the RLS intensity of the system is enhanced. This indicates that there is interaction between Eu^3+^ and nicotinic acid, and a complex forms. After the addition of nucleic acids, the RLS intensity of Eu^3+^ complex is greatly enhanced, which indicates that there is a further interaction between fsDNA and the Eu^3+^ complex forming a larger complex, resulting in the enhancement of the RLS intensity of the system. It is considered that Eu^3+^ complex is positively charged, and can bind with phosphate groups in the nucleic acids through electrostatic force and thus form a larger Eu^3+^-nicotinic acid-fsDNA complex. 

## Conclusions

We have found that nucleic acids could greatly enhance the RLS intensity of Eu^3+^-nicotinic acid. The enhanced intensity is in proportion to the concentration of fsDNA in the range of 7×10^-8^-1×10^-5 ^g∙mL^-1^, with a detection limit of 2×10^-8^g∙mL^-1^. The synthetic and actual samples are satisfactorily determined. The interaction mechanism is also studied. It is considered that nucleic acid can bind with the complex of Eu^3+^-nicotinic acid through electrostatic attraction and form a large Eu^3+^-nicotinic acid-nucleic acid complex. The study is helpful for us to explore the interaction mechanism between metal ion complex and nucleic acid.

## Experimental

### General

*Chemicals:* Stock solutions of RNA (1.0×10^-4 ^g∙ml^-1^) and DNA (1.0×10^-4 ^g∙ml^-1^) were prepared by dissolving commercial yeast RNA (yRNA) (Institution Biochemistry, Chinese Academy of Sciences) and fish sperm DNA (fsDNA, Sigma Co., USA) in 0.05 M sodium chloride solution. These stocks needed to be stored at 0-4^ o ^C. The working solution was prepared by diluting the stock solution to the proper concentration. The purity of DNA was checked by measuring the ratio of the absorbance of 260 nm to that of 280 nm. Stock standard solution of Eu^3+ ^(2.0 x 10^-3 ^M) was prepared by dissolving 0.3520 g of the corresponding europium oxide (99.99 %, Shanghai Yuelong Chemical Plant, P.R. China) in hydrochloric acid (10 mL) and then diluting to 100 mL with water. Stock standard solution (1.0 x 10^-3^ M) of nicotinic acid (Sinopharm Chemical Reagent Co., Ltd, P.R. China) was made by dissolving nicotinic acid (0.0308 g) in water (250 mL). A 0.05 M Tris–HCl buffer solution was prepared by dissolving appropriate Tris in water and adjusting the pH with HCl. All the chemicals used were of analytical reagents grade and doubly deionized water was used throughout.

*DNA extraction*: The extraction process of DNA in cucumber is as follows: after the cucumber sample is carefully ground in liquid nitrogen, a 0.2000 g sample is accurately weighed and dissolved in cell extraction solution (600 μL), then incubated in a water-bath at 65 °C for 20 min. The same volume of CHCl_3_ is then added and mixed well, and the mixture then centrifuged for 10 min at 12,000 rpm and the supernatant taken. This is repeated until no protein can be seen. The supernants are combined and the same volume of isopropyl alcohol is added and the DNA is then precipitated in an ice-bath for 30 min and centrifuged for 10 min at 12,000 rpm. The supernatant is discarded before washing the DNA with ethanol (70%). The extracted DNA is dissolved in 0.05 M sodium chloride solution (1.5 mL) and stored at 0-4°C.

*Apparatus:* All RLS spectra were recorded on a LS-55 spectrofluorimeter (PE) in a 1 cm quartz cell. The ultraviolet absorption spectra were performed with a UV-4100 spectrophotometer (Hitachi, Japan) equipped with 1 cm quartz cells. All pH measurements were made with a Delta 320-s pH meter (Mettler Toledo, Shanghai).

*Analytical procedure:* To a dry 10 mL test-tube, solutions were added as the following order: nicotinic acid (0.5 mL), definite standard nucleic acid (or sample solution), Eu^3+^ (1.4 mL, 2.00×10^-3^ M) and Tris-HCl buffer (pH 8.1, 0.55 mL). The mixture was diluted to 10.0 mL with water and allowed to stand for 15 min. All RLS spectra were obtained by scanning simultaneously the excitation and emission monochromators (namely, λ = 0 nm) from 270 to 580 nm. The intensity of RLS was measured at λ = 387.0 nm in a 1.00 cm quartz fluorescence cell with slit width of 10 nm for both the excitation and emission. The enhanced RLS intensity of the Eu^3+^–nicotinic acid system by nucleic acids is represented as ΔI_R_ = I_R _- I_R_^0^. Here I_R_ and I_R_^0^ are the RLS intensity of the system with and without nucleic acids, respectively.
